# The risk of nonsyndromic cleft lip with or without cleft palate and *Vax1* rs7078160 polymorphisms in southern Han Chinese

**DOI:** 10.1016/j.bjorl.2020.08.007

**Published:** 2020-10-11

**Authors:** Qian Wang, Sichao Sun, Qinggao Song, Huan Hu, Jiaxing An, Jianguo Liu

**Affiliations:** a Oral Disease Research Key Laboratory of Guizhou Tertiary Institution, School of Life Science, Zunyi Medical University, Zunyi, China; b Microbial Resources and Drug Development Key Laboratory of Guizhou Tertiary Institution, School of Life Science, Zunyi Medical University, Zunyi, China; c School of Stomatology, Zunyi Medical University, Zunyi, China; d Affliated Hospital of Zunyi Medical University, Zunyi, China

**Keywords:** Cleft lip, Cleft palate, Single nucleotide polymorphisms, *Vax1*, rs7078160

## Abstract

**Introduction:**

Non-syndromic cleft lip with or without cleft palate is a common worldwide birth defect due to a combination of environmental and genetic factors. Genome-wide association studies reported the rs7078160 of *Vax1* is closely related to non-syndromic cleft lip with or without cleft palate in European populations. The following studies showed the same results in Mongolian, Japanese, Filipino, Vietnamese populations etc. However, conflicting research had been reported in Chinese population,

**Objective:**

The aim of this study was to investigate the association between the rs7078160 polymorphism and non-syndromic cleft lip with or without cleft palate in Southern Chinese patients.

**Methods:**

In this study, we investigated the polymorphism distribution of rs7078160 in 100 complete patient trios (39 patients with non-syndromic cleft lip and palate; 36 patients with non-syndromic cleft lip only; 25 had non-syndromic cleft palate only; and their parents) from Southern ethnic Han Chinese. 60 healthy trios were selected as control. Polymerase chain reaction and Sanger sequencing were used to genotype rs7078160 in *Vax1*; both case–control and family-based associations were analyzed.

**Results:**

The case–control analyses revealed the rs7078160 polymorphism was significant, associated with non-syndromic cleft lip with or without cleft palate (*p* = 0.04) and non-syndromic cleft lip and palate (*p* = 0.01), but not associated with non-syndromic cleft lip only and non-syndromic cleft palate only patients. The genotype composition of rs7078160 comprises mutated homozygous AA, heterozygous AG and wild homozygous GG. Cases with AG + AA genotypes compared with GG homozygotes showed an increased risk of non-syndromic cleft lip with or without cleft palate (*p* = 0.04, OR = 2.05, 95% CI: 1.01–4.16) and non-syndromic cleft lip and palate (*p* = 0.01, OR = 3.94, 95% CI: 1.34–11.54). In addition, we did not detect any transmission-disequilibrium in rs7078160 (*p* = 0.68).

**Conclusion:**

This study suggests that rs7078160 polymorphism is a risk factor of non-syndromic cleft lip with or without cleft palate, and *Vax1* is strongly associated with non-syndromic cleft lip with or without cleft palate in Southern Chinese Han populations.

## Introduction

Non-syndromic cleft lip with or without cleft palate (NSCL/P) is one of the most common worldwide birth defect, with an incidence of 1.2 in 1000 live births.^1^ In China, the prevalence of NSCL/P is higher than that in other racial and ethnic populations (1.48–3.27 in 1000 live births).^2^, ^3^ Currently, with the development of plastic surgery, more patients with cleft lip or palate can be repaired by surgery^4^; however, they may still suffer from a handful of problems such as craniofacial deformation and ambiguous speech. Not only that, although international charities such as the Smile Train have provided support for many patients, the therapy of NSCL/P may be a heavy economic burden for some families, especially in the developing countries. Hence, clarifying the etiologies of NSCL/P is very important for disease diagnosis and prevention.

The etiologies and pathogenesis of NSCL/P are complex, including both genetic and environmental factors or their combination effort.^3^ In recent years, with the development of high-throughput sequencing technology and statistical methodology, Genome-Wide Association Studies (GWAS) have been made to identify candidate genes involved in the development of NSCL/P.^5^ GWAS evidences have identified dozens of potential causative loci and genes of NSCL/P, such as *Irf6*, *Mafb*, *Grhl3*, *Abca4*, *Nog*, *Spry2*, *Tpm1*, 8q21.3, *Crebbp* and *Vax1*.^6^, ^7^, ^8^, ^9^, ^10^ However, in the subsequent replications, few of those loci/genes showed consistently positive correlations across all studies. In fact, for more candidate genes, inconsistent results were reported in populations from different races and regions. *Vax1* is one of such genes.

A GWAS conducted by Mangold et al. identified a new loci rs7078160 associated with NSCL/P at 10q25 in European population.^11^ Subsequently, Beaty et al. conducted another GWAS study in European and Asian groups, their founding confirmed rs7078160 of *Vax1* was correlated with NSCL/P.^6^ Following studies in Mongolian, Japanese, Filipinos and Vietnamese populations supported above results.^12^, ^13^, ^14^ However, conflicting research has been reported in the Chinese population. A study by Li et al. found rs7078160 of *Vax1* was not associated with NSCL/P in northern Chinese Han population.^15^ In this study, we first investigated the correlation between *Vax1* rs7078160 polymorphisms and the risk of NSCL/P in the Southern Han Chinese population. Considering that complete trio samples could provide more reliable data than case–control samples, case/control-parents design was used in this study, so we could detect the genotypic and allelic distributions of target single nucleotide polymorphism (SNP) while analyzing the parental origin effects.

## Methods

### Subjects

The study was conducted in the Department of Oral and Maxillofacial Surgery, affiliated with the Stomatological Hospital of Zunyi Medical University (Zunyi, Guizhou, China) from 2016 to 2019. One hundred NSCL/P patients (39 patients with non-syndromic cleft lip and palate, NSCLP; 36 patients with non-syndromic cleft ip only, NSCLO; and 25 with non-syndromic cleft palate only, NSCPO) and their parents were recruited as subjects. Sixty healthy matched trios were selected as the control. The protocol of the study was approved by the Institutional Ethics Committee of Zunyi Medical University (ZMU-HG1/Sept/25/16 date: 11/01/2016). Informed consents were collected from the parents or their legal guardians.

Clinical assessment was conducted by experienced oral and maxillofacial surgeons, to exclude the syndromic CL/P patients associated with any other congenital disabilities. All NSCLP, NSCLO and NSCPO cases were unilateral and none of the patients had affected relatives; patients with family history of genetic disease were also excluded. Healthy volunteers without a family history of cleft lip and/or palate or other abnormality were chosen as controls. All cases and controls were Chinese Han population, with the same age and gender distribution, and lived in Southern China for at least three generations. Three milliliters peripheral venous blood was collected from each subject and stored at −80 °C.

### DNA extraction, PCR ampliﬁcation and sequencing

**Figure 1:**
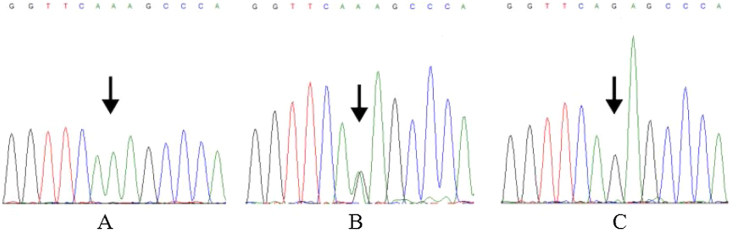
DNA sequencing results of rs7078160. (A) Mutated homozygous genotype AA. (B) Heterozygous genotype AG. (C) Wild homozygous genotype GG.

Genomic DNA was extracted from blood sample by TIANamp Blood DNA kit (Tiangen Biotech, Beijing, China). The specific rs7078160 primers were designed according to *Vax1* reference sequences in NCBI (National Center for Biotechnology Information, Bethesda, MD, USA) by Primer 5.0 software (Premier Biosoft International, Palo Alto, CA, USA). The sequence of forward primer was 5′ TGGGAAGTGGGTGAGATGGA 3′, reverse primer was 5′ ATTGGGCGGACCCAGTAAAG 3′. All PCR ampliﬁcations were performed on a Gene Amp PCR system 9700 thermal cycler (Applied Biosystems, Foster City, CA, USA). The reaction mixture consisted of 10–30 ng of genomic DNA, 1 μL of each primer (10 μmoL L^−1^), 4 μL dNTPs (2.5 mmoL L^−1^ of each nucleobase), 5 μL 10× Buffer (with MgCl_2_) and 1.0 U of Taq polymerase (TaKaRa, Dalian, China), add ddH_2_O to 50 μL volume. The PCR amplification program was: (1) denaturing (94 °C, 4 min); (2) 34 cycles including denaturing (94 °C, 30s), annealing (58 °C, 30 s), extension (72 °C, 40 s), (3) final extension (72 °C, 5 min). PCR product was detected by agarose gel electrophoresis and puriﬁed. Sequencing was conducted by BGI (Beijing Genomics Institute, Beijing, China). We aligned and edited sequences using DNA sequencing software Chromas 2.6.6 (Technelysium, Queensland, Australia) and MEGA X.^16^ The DNA sequencing results of rs7078160 were shown in Figure 1.

### Statistical methods

The genotype and allele frequencies were summarized in the cases (total group, NSCLP, NSCLO and NSCPO subgroup) and the control. The Hardy–Weinberg equilibrium (HWE) states that both allele and genotype frequencies in a random mating population remain constant, in order to demonstrate the genetic stability of our subjects, HWE was assessed according to the genotype frequency. Results were tested with a Chi-square test in SPSS 16.0 (SPSS Inc., Chicago, IL, USA). In case-parents’ trios, the transmission disequilibrium test (TDT) was conducted by statistical analysis for genetic epidemiology (S.A.G.E.) software package. TDT is a family-based test which offers a powerful way to test the transmission of target alleles from heterozygous parents to affected offspring. The odds ratio (OR) and 95% confidence intervals (95% CI) were also calculated, OR > 1 signified an increased risk of NSCL/P. All data were tested by two-sided *p*-value, *p* < 0.05 were accepted as significant.

## Results

The Hardy–Weinberg equilibrium analysis revealed all the subjects showed a genetic equilibrium at rs7078160 (Table 1). The distribution of the rs7078160 variant genotypes and alleles in both case groups and controls are presented in Table 1. The proportions of genotypes were 21% AA, 57% AG, 22% GG in cases and 11.7% AA, 51.7% AG, 36.7% GG in controls. The G allele frequency was 50.5% in cases and 62.5% in controls. The allele frequencies of rs7078160 in the total case group (*p* = 0.04) and NSCLP subgroup (*p* = 0.01) were substantially different when contrasted to the control group, however, the allele frequencies in the NSCLO (*p* = 0.19) and NSCPO subgroup (*p* = 0.43) were not significant when compared with the control group (Table 1).

**Table 1:** Genotypic and allelic distributions of rs7078160 in NSCL/P patients and controls (^a^*p* < 0.05).

Genotype	Total	NSCLP	NSCLO	NSCPO	Controls
*Test for HWE*					
Chi-square	1.96	3.41	0.47	0.02	0.63
*p*-value	0.16	0.06	0.49	0.90	0.43

*Genotype distribution*					
AA (*n*, %)	21 (21)	9 (23.1)	7 (19.4)	5 (20.0)	7 (11.7)
AG (*n*, %)	57 (57)	25 (64.1)	20 (55.6)	12 (48.0)	31 (51.7)
GG (*n*, %)	22 (22)	5 (12.8)	9 (25.0)	8 (32.0)	22 (36.7)
Chi-square	4.99	7.48	1.95	1.02	
*p*-value	0.08	0.02^a^	0.38	0.60	

*Allele frequency*					
A (*n*, %)	99 (49.5)	43 (54.4)	34 (47.2)	22 (44.0)	45 (37.5)
G (*n*, %)	101 (50.5)	35 (44.3)	38 (52.8)	28 (56.0)	75 (62.5)
Chi-square	4.36	5.95	1.76	0.62	
*p*-value	0.04^a^	0.01^a^	0.19	0.43	

NSCLP, non-syndromic cleft lip and palate; NSCLO, non-syndromic cleft lip only; NSCPO, non-syndromic cleft palate only.

The OR and 95% CI between AG + AA and GG for rs7078160 were also significant difference in the total case group (*p* = 0.04, OR = 2.05, 95% CI: 1.01–4.16) and the NSCLP subgroup (*p* = 0.01, OR = 3.94 95% CI: 1.34–11.54); the results were not significant in the NSCLO (*p* = 0.24) and NSCPO subgroup (*p* = 0.68) (Table 2). The transmission disequilibrium test was conducted for trios with heterozygote genetic structure, and no statistically signiﬁcant transmitted disequilibrium was detected in rs7078160 (*p* = 0.68) (Table 3).

**Table 2:** Results of association tests with rs7078160 in non-syndromic cleft lip with or without cleft palate (NSCL/P) (^a^*p* < 0.05).

	Variable					
	AG + AA	GG	Chi-square	*p*-value	Odds ratio	95% CI
Controls	38	22				
Total	78	22	4.05	0.04^a^	2.05	1.01–4.16
Non-syndromic cleft lip and palate (NSCLP)	34	5	6.78	0.01^a^	3.94	1.34–11.54
Non-syndromic cleft lip only (NSCLO)	27	9	1.40	0.24	1.74	0.69–4.35
Non-syndromic cleft palate only (NSCPO)	17	8	0.17	0.68	1.23	0.46–3.31

**Table 3:** Results of the transmission disequilibrium test.

rs7078160	Transmitted	Not transmitted	Total
	A	G	
A	27	50	77
G	45	22	67
Total	72	72	144
Chi-square	0.26		
*p*-value	0.68		

## Discussion

The important etiological role of rs7078160 was first reported by Mangold et al. in a genome-wide association study of European NSCL/P populations.^11^ The top susceptive NSCL/P SNP in 10q25 was rs7078160 in *Vax1* (Ventral Anterior Homeobox 1). *Vax1* was a crucial gene for the development of the basal forebrain and visual system in animal experiments, and it also participated in the rostral formation of vertebrates.^17^ Slavotinek et al. reported that *Vax1* mutation is associated with human cleft lip and palate, absence of the pineal gland, corpus callosum agenesis, hippocampal malformations and small optic nerves.^18^

To date, several studies have confirmed the association between rs7078160 and NSCL/P in different racial and ethnic populations, including Japanese, Polish, central Africans and southeast Asian populations.^12^, ^13^, ^14^ However, inconsistent results were found in Chinese, Kenyan and another Polish cases.^15^, ^19^, ^20^ In Chinese, research of Li et al. fail to identify the risk of *Vax1* in NSCL/P in northern Chinese population.^15^ However, recently a study conducted by Zhang et al. found the *Vax1* gene was a risk factor for NSCL/P in western Han Chinese population.^5^ Apart from the geographical difference, the different studies method might be an explanation; the former studies used case–control design, while Zhang et al. adopted a case-parent trio design.^5^ In this study, we chose the Southern Chinese Han population as the object. The allele frequencies of rs7078160 in the NSCLP group were substantially different when contrasted to the control group (*p* = 0.02, respectively) (Table 1). OR and 95% CI between AG + AA and GG showed a significant difference in the total case group (*p* = 0.04, OR = 2.05, 95% CI: 1.01–4.16) and NSCLP subgroup (*p* = 0.01, OR = 3.94, 95% CI: 1.34–11.54), suggested that allele A of rs7078160 may be a risk factor for NSCL/P, but not associated with NSCLO and NSCPO (Table 2). The TDT test did not detect significant transmitted disequilibrium in rs7078160 (*p* = 0.68) (Table 3). This result was conflicting with the previous NSCL/P studies by Li et al., in which they failed to find an association of *Vax1* and NSCL/P in northern Chinese Han population.^15^ The inconsistency might be due to the geographical difference and different sampling design. The complete case-parent trio might provide more comprehensive genetic information compared to the case–control design.

Collectively, this study has demonstrated that rs7078160 is involved in the development of NSCL/P in Southern Han Chinese. Our study provided additional evidence for the etiology of *Vax1* in NSCL/P of Chinese Han population. However, our study still has several potential limitations. First, we focused on only one SNP rs7078160; the study of more candidates with SNP could enhance the significance of our study. Second, due to the difficulties in collecting the complete trio samples, the number of cases and control trios were relatively limited. To increase the number of the subjects would strengthen our results. Thirdly, the environmental interactions and maternal factors during pregnancy were not evaluated in our study. Further efforts are still needed to clarify the deeper association between heredity and environment.

## Conclusion

This study had revealed that rs7078160 was involved in the development of NSCL/P in Southern Chinese Han population, demonstrating *Vax1* might be associated with NSCL/P. Those findings could be helpful in extending our knowledge about NSCL/P etiology.

## Conflicts of interest

The authors declare no conflicts of interest.
